# Control of pathogenic bacteria using marine actinobacterial extract with antiquorum sensing and antibiofilm activity

**DOI:** 10.1186/s13104-023-06580-z

**Published:** 2023-11-02

**Authors:** Marco Wijaya, Dea Delicia, Diana Elizabeth Waturangi

**Affiliations:** Faculty of Biotechnology, Atma Jaya Catholic University of Indonesia, Jenderal Sudirman 51 Street, South Jakarta, DKI Jakarta 12930 Indonesia

**Keywords:** Actinobacteria, Biofilm formation, Biofilm-forming bacteria, *Chromobacterium violaceum*, Quorum sensing, Quorum quenching

## Abstract

**Objective:**

The objectives of this research were to screen the anti-quorum sensing and antibiofilm activity of marine actinobacteria, isolated from several aquatic environments in Indonesia against several pathogenic bacteria, such as *Staphylococcus aureus*, *Bacillus cereus*, *Enterococcus faecalis*, *Vibrio cholerae*, *Salmonella* Typhimurium, and *Pseudomonas aeruginosa.*

**Results:**

Ten out of 40 actinobacteria were found to have anti-quorum sensing activity against wild-type *Chromobacterium violaceum* (ATCC 12472); however, the validation assay showed that only eight of 10 significantly inhibited the quorum sensing system of *Chromobacterium violaceum* CV026. The crude actinobacteria extracts inhibited and disrupted biofilm formation produced by pathogens. The highest antibiofilm inhibition was discovered in isolates 11AC (90%), 1AC (90%), CW17 (84%), TB12 (94%), 20PM (85%), CW01 (93%) against *Staphylococcus aureus*, *Bacillus cereus*, *Enterococcus faecalis*, *Vibrio cholerae*, *Salmonella* Typhimurium, and *Pseudomonas aeruginosa*, respectively. The highest biofilm destruction activity was observed for isolate 1AC (77%), 20PM (85%), 16PM (72%), CW01 (73%), 18PM (82%), 16PM (63%) against *Staphylococcus aureus*, *Bacillus cereus*, *Enterococcus faecalis*, *Vibrio cholerae, Salmonella* Typhimurium, and *Pseudomonas aeruginosa*, respectively. Actinobacteria isolates demonstrated promising anti-quorum and/or antibiofilm activity, interfering with the biofilm formation of tested pathogens. Appropriate formulations of these extracts could be developed as effective disinfectants, eradicating biofilms in many industries.

**Supplementary Information:**

The online version contains supplementary material available at 10.1186/s13104-023-06580-z.

## Introduction

Antimicrobials have been very effective in arresting pathogens’ growth in many circumstances [1, 2]. Unfortunately, rampant usage and poor patient compliance have accelerated antimicrobial resistance in bacteria. Infection of antimicrobial-resistant (AMR) bacteria can be life-threatening. Moreover, AMR bacteria such as *S. aureus* and *S.* Typhimurium, can produce biofilms [3, 4]. In illnesses attributed to biofilm-forming bacteria, extracellular matrix developed, increasing their resistance to chemical treatments up to 1000-fold greater than its planktonic form [5].

The uncontrolled growth of biofilm-forming bacteria causes an enormous number of outbreaks and poses global risks by either infection, intoxication, or toxicoinfection. Therefore, there is a growing need to identify new solutions to control biofilm-forming pathogens [6].

Quorum sensing (QS) is the cell-to-cell communication activity of bacteria using autoinducers that regulates virulence factor and biofilm formation [6, 7]. Consequently, inhibiting QS activity might initiate biofilms’ eradication. Without QS activity, bacterial aptitude to protect themselves will decline drastically.

Actinobacteria are fungi-like Gram-positive bacteria, known as the leading producer of secondary metabolites, including novel anti-QS and biofilm inhibitors [8]. However, the research has been only minimally explored. This research aims to screen anti-QS compounds from actinobacteria using wild-type *Chromobacterium violaceum* and *Chromobacterium violaceum* CV026 as QS indicators and quantify their antibiofilm activity against biofilm-forming pathogens.

## Main text

### Methods

#### Bacterial cultivation

Actinobacteria isolates were retrieved from previous studies [9, 10]. Actinobacteria were cultured on yeast malt extract agar (YMEA; 4 g yeast extract, 10 g malt extract, 4 g glucose, 2% agar) and incubated at 28 °C for 7 days. Wild-type *Chromobacterium violaceum* (ATCC 12472) and *Chromobacterium violaceum* CV026 as QS indicators were cultivated on Luria agar (LA; Oxoid) and incubated at 28 °C for 24 h. *C. violaceum* CV026 is a pigment-negative strain due to mutation in *CviI*, encoding AHL synthase for autoinducer production. However, in the presence of exogenous AHL, *C. violaceum* CV026 will express QS-mediated responses leading to pigment production [11]. Biofilm-forming bacteria *B. cereus* ATCC 14579, *S. aureus* ATCC 29213, *E. faecalis* ATCC 33186, *P*. *aeruginosa* ATCC 27853, *S.* Typhimurium, and *V*. *cholerae* were cultivated onto LA, incubated at 37 °C for 24 h. These pathogens can initiate biofilm formation via QS activity [3, 4, 12–15].

#### Primary screening of anti-quorum sensing activity

Primary screening was carried out using an overlay agar method [16] with modifications. Actinobacteria isolates were inoculated onto YMEA and incubated at 28 °C for 3 days. Wild-type *C. violaceum* (OD_600_ = 0.132, 100 µL) was mixed with 2 mL semi-solid (0.75%) LA and poured atop YMEA plates. The plates were incubated at 28 °C for 24 h. Inhibited violacein production around actinobacteria isolates signifies anti-QS activity.

#### Preparation of crude extract

The extract was obtained using liquid-liquid extraction. Actinobacteria isolate was grown into tryptic soy broth supplemented (Oxoid) with glucose (1% w/v) at 28 °C and 125 rpm for 7 days. The cultures were centrifuged at 7800×*g* for 15 min. The supernatant was mixed with ethyl acetate (1:1) and shaken at 150 rpm for 24 h. The solvent was collected and evaporated using a rotary evaporator then dried using a vacuum oven. Crude extract was mixed in 1% v/v dimethyl sulfoxide (DMSO) to provide 5, 10, and 20 mg/mL concentrations [17, 18].

#### Antimicrobial assay

Antimicrobial assays were performed using the agar well diffusion method [19] with modifications. Pathogens (OD_600_ = 0.132) were spread onto brain heart infusion agar (BHIA, Oxoid). Wells were created and filled with the extract (50 µL, 5 and 10 mg/mL). DMSO (1% v/v) was used as the negative control, and streptomycin (10 mg/mL) was used as the positive control.

Incubation at 37 °C for 24 h revealed clear zones, indicating antibacterial activity.

#### Secondary screening of anti-quorum sensing activity

Wild-type *C. violaceum* (OD_540_ = 0.132) was spread onto BHIA plates. Wells were created and filled with the extract (50 µL, 5 and 10 mg/mL). DMSO (1% v/v) was used as the control [20]. The plates were incubated at 28 °C for 24 h. Inhibited violacein production indicated anti-QS activity [21].

#### Quantification of antibiofilm activity

The antibiofilm activity was categorized as inhibition or elimination. To detect inhibition activity, pathogens (OD_600_ = 0.132, 100 µL) cultivated into Brain Heart Infusion Broth supplemented with glucose (2% w/v), and extracts (5 and 10 mg/mL, 100 µL) were transferred to the 96-well microplate. Biofilm inhibition activity was quantified after 24 h. For the elimination activity assay, another 96-well plate with bacterial culture was incubated at 37 °C for 24 h. After biofilms were attached, extract was added and incubated for 24 h. Each pathogenic culture was used as the positive control, while sterile BHIB was used as the negative control.

After incubation, planktonic cells and media were discarded. Adherent cells were rinsed twice with sterile water and stained with crystal violet (0.4% w/v) for 30 min. The microplate was rinsed twice and air-dried for 5 min. Subsequently, 200 µL of ethanol was mixed. Absorbance was determined at 595 nm [22]. Antibiofilm activity was calculated using this equation:

$$ \% \text{Activity} \frac{{\text{OD} \;\text{Control} - \text{OD}\; \text{Treated}}}{{\text{OD}\; \text{Control}}} \times 100 \%.$$

#### Validation of Quorum sensing inhibition

*Chromobacterium violaceum* CV026 (OD_600_ = 0.132, 100 µL) was mixed with 100 µL of extract (final concentration 10 mg/mL) and 1 µL hexanoyl-l-homoserine-lactone (HHL, final concentration 100 mM, Sigma-Aldrich) diluted in acidified ethyl acetate (0.1% v/v acetic acid). Incubation at 28 °C for 24 h followed. Mixture without extract served as a positive control. After incubation, test tubes were centrifuged at 1000 rpm for 15 min. The pellet was mixed with 1 mL DMSO (1%v/v) and centrifuged at 1000 rpm for 15 min. The supernatant’s absorbance was measured at 540 nm [18]. Furthermore, to investigate whether the main compounds in the extract were protein-based, the determination was carried out using proteinase-K. Each extract was treated with proteinase-K (100 µg/mL) for 2 h incubation at 37 °C and subjected to high-temperature treatment at 95 °C for 1 h. The treated extract was used for the validation assay. Total violacein produced by *C. violaceum* CV026 was compared with the untreated batch [23].

#### Statistical analysis

Data were compared using a one-way ANOVA based on a confidence level at 95% and Tukey HSD post-hoc analysis.

##### Examination of biofilm formation by scanning electron microscope (SEM)

*Bacillus cereus* and *Salmonella* Typhimurium culture (OD_600_ = 0.132) were spotted onto sterile cover glass within sterile Petri dishes and incubated at 37 °C for 24 h to form mature biofilms. After incubation, actinobacterial extract (100 µL, 10 mg/mL) was added and re-incubated for 24 h. The results were investigated using SEM at Dexa Laboratory of Biomolecular and Science [24].

### Results

#### First screening of anti-quorum sensing activity

The results show that 10 out of 40 actinobacteria isolates had anti-QS activity (Additional file 1: Illustration S1). Those isolates were extracted and continued to the next step (Additional file 1: Illustration S2).

#### Antibacterial activity assay

Ten actinobacterial extracts showed no antibacterial activity against *S. aureus*, *B. cereus*, *E. faecalis*, *P. aeruginosa*, and *S.* Typhimurium, but 3 of the 10 showed antibacterial activity against *V. cholerae*. Those extracts were 15PM, 18PM, and 20PM. Streptomycin as the positive control inhibited the growth of all pathogens. In contrast, DMSO as the negative control showed no antibacterial activity (Additional file 1: Illustration S3).

#### Secondary screening of anti-quorum sensing activity

Ten actinobacterial extracts in 5 and 10 mg/mL concentrations still had anti-QS activity. It was demonstrated by the absence of violacein pigment around the wells (Additional file 1: Illustration S4).

#### Anti-quorum sensing activity validation test and protein characterization

**Fig. 1:**
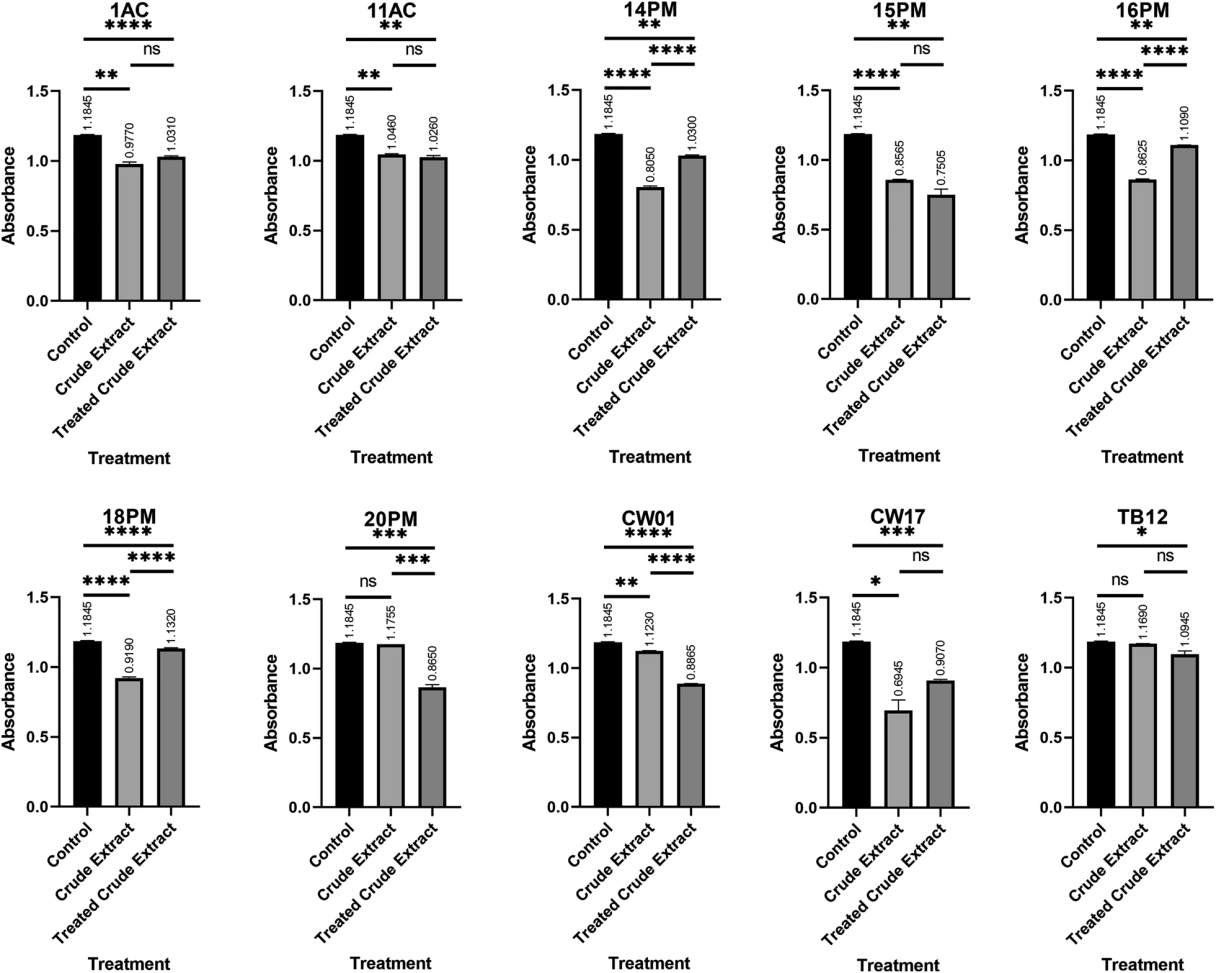
Absorbance of total violacein produced by *C. violaceum* CV026

All treatments yielded lower absorbance than the control (Fig. 1). However, only 8 out of 10 extracts (10 mg/mL) demonstrated statistically significant anti-QS activity against *C. violaceum* CV026: 1AC, 11AC, 14PM, 15PM, 16PM, 18PM, CW01, and CW17. Treated extract of all isolates showed anti-QS activity distinct from the control. Increased absorbance suggests interference with anti-QS compounds due to proteolytic and high-temperature treatments, while lower values indicate heightened anti-QS activity.

#### Biofilm inhibition and elimination assay

Crude extracts showed diverse antibiofilm activity in inhibiting and eliminating bacterial biofilms (Table 1).

**Table 1:** Antibiofilm activity of actinobacterial crude extracts against biofilm-forming bacteria

Pathogen	Antibiofilm activity	Crude extract (mg/mL)	% Activity									
			1AC	11AC	14PM	15PM	16PM	18PM	20PM	CW01	CW17	TB12
SA		5	65 ± 1.57	87 ± 0.80^a^	78 ± 0.56	43 ± 0.97	64 ± 3.51	46 ± 2.12	55 ± 2.36	76 ± 2.55	74 ± 0.68	44 ± 1.00
		10	90 ± 0.38^a^	90 ± 0.61^a^	79 ± 1.84	66 ± 1.96	75 ± 0.22	74 ± 0.47	76 ± 1.64	77 ± 0.81	75 ± 0.07	62 ± 0.44
BC		5	89 ± 0.27^a^	85 ± 0.56	76 ± 0.23	78 ± 1.04	84 ± 1.26	57 ± 1.04	86 ± 0.93	84 ± 0.25	85 ± 1.21	81 ± 0.91
		10	90 ± 1.46^a^	87 ± 1.34	76 ± 1.34	83 ± 1.55	87 ± 1	69 ± 0.31	88 ± 1.01	87 ± 0.63	85 ± 1.43	84 ± 1.24
EF		5	49 ± 3.53	70 ± 0.22	12 ± 2.84	36 ± 2.88	43 ± 1.93	15 ± 4.00	27 ± 1.60	76 ± 2.47	83 ± 0.62^a^	33 ± 4.84
	Inhibition	10	82 ± 1.00	71 ± 2.37	34 ± 1.27	71 ± 0.12	77 ± 0.57	40 ± 1.24	50 ± 1.90	83 ± 0.89	84 ± 1.75^a^	81 ± 1.07
VC		5	93 ± 0.23	89 ± 0.34	90 ± 2.72	X	93 ± 0.82	X	X	92 ± 1.01	92 ± 0.30	94 ± 0.50^a^
		10	76 ± 1.58	79 ± 3.59	84 ± 1.75^a^	X	82 ± 2.15	X	X	83 ± 1.67	70 ± 1.27	58 ± 1.61
ST		5	76 ± 0.34	83 ± 0.26	84 ± 0.23	80 ± 0.90	81 ± 1.53	81 ± 0.46	85 ± 0.33^a^	80 ± 2.82	81 ± 0.38	81 ± 1.36
		10	56 ± 3.02	63 ± 4.66	50 ± 1.49	54 ± 1.34	27 ± 0.27	76 ± 0.51^a^	59 ± 0.13	63 ± 2.62	66 ± 3.62	25 ± 0.92
PA		5	29 ± 2.21	70 ± 2.77	49 ± 1.98	38 ± 2.13	50 ± 1.13	39 ± 3.25	37 ± 4.07	47 ± 0.19	54 ± 0.75^a^	42 ± 4.32
		10	83 ± 4.32	82 ± 2.00	86 ± 1.82	91 ± 4.35	85 ± 2.14	81 ± 3.18	89 ± 1.63	93 ± 2.34^a^	87 ± 3.97	37 ± 4.55
SA		5	62 ± 2.05^a^	32 ± 2.16	41 ± 1.95	38 ± 0.45	40 ± 1.75	38 ± 1.24	42 ± 4.78	45 ± 1.85	50 ± 2.91	37 ± 4.94
		10	77 ± 0.70^a^	49 ± 1.59	54 ± 0.86	74 ± 0.78	59 ± 0.88	65 ± 1.62	62 ± 1.62	60 ± 1.08	59 ± 1.61	61 ± 1.00
BC		5	61 ± 1.38	67 ± 1.22	64 ± 1.11	67 ± 0.42	65 ± 1.33	67 ± 2.23	75 ± 1.33^a^	72 ± 1.14	73 ± 0.91	67 ± 0.17
		10	84 ± 1.94	80 ± 1.31	78 ± 0.36	68 ± 1.12	84 ± 2.16	75 ± 1.88	85 ± 0.97^a^	83 ± 1.12	74 ± 1.74	79 ± 0.81
EF		5	43 ± 0.96	44 ± 4.23	50 ± 3.19	47 ± 3.35	47 ± 1.82	45 ± 4.90	46 ± 4.48	50 ± 0.68	52 ± 1.55^a^	43 ± 1.40
	Destruction	10	58 ± 3.15	63 ± 1.64	55 ± 2.39	70 ± 1.43	72 ± 2.03^a^	70 ± 1.95	65 ± 2.40	52 ± 0.86	63 ± 0.93	45 ± 1.69
VC		5		52 ± 1.05		X	71 ± 0.70	X	X	73 ± 0.74^a^	71 ± 0.92	70 ± 0.61
		10	11 ± 2.46	48 ± 2.72	62 ± 0.75^a^	X	13 ± 4.33	X	X	43 ± 1.64	52 ± 2.70	43 ± 3.06
ST		5	57 ± 0.85	43 ± 2.10	54 ± 0.50	34 ± 1.60	29 ± 0.36	58 ± 0.58	51 ± 0.45	66 ± 0.19	73 ± 0.51^a^	64 ± 0.38
		10	56 ± 1.71	20 ± 1.25	72 ± 1.46	54 ± 0.69	47 ± 3.29	82 ± 014^a^	64 ± 0.30	79 ± 0.37	78 ± 0.82	42 ± 1.95
PA		5	11 ± 1.52	53 ± 0.97	29 ± 1.68	54 ± 4.07	63 ± 4.67^a^	17 ± 3.27	59 ± 2.92	38 ± 2.82	26 ± 4.79	58 ± 2.19
		10	43 ± 0.92	45 ± 1.09	41 ± 3.22	57 ± 2.95	42 ± 0.82	50 ± 2.79	55 ± 2.03	50 ± 0.71	55 ± 2.73	50 ± 0.54

*Staphylococcus aureus*ATCC 29213; *Bacillus cereus*ATCC 14579; *Enterococcus faecalis*ATCC 33186, *Vibrio cholerae, Salmonella*Typhimurium, *Pseudomonas aeruginosa*ATCC 27853

^a^Highest activity, X Have antimicrobial activity, −No activity

#### Examination of biofilm formation by scanning electron microscope

The result showed the topographical of *B. cereus* and *S.* Typhimurium biofilms before and after treatment with actinobacterial extract. Considerable morphological changes occurred in bacterial biofilms.

### Discussion

The primary screening of anti-QS activity showed that 10 out of 40 isolates demonstrated anti-QS activity. In adapting to competitive environments, actinobacteria produce secondary metabolites such as anti-QS and anti-biofilms [25]. No clear zones were observed in the antimicrobial assay, indicating negative-antimicrobial activity, except in *V. cholerae*. Of the 10 actinobacteria isolates, three (15PM, 18PM, and 20PM) inhibited the growth of *V. cholerae*. Those isolates were excluded in the *V. cholerae* antibiofilm assay. Extract in 5 and 10 mg/mL concentrations inhibited the QS system of wild-type *C. violaceum*, as seen from lower pigment production around the wells. From the validation assay, only 8 of 10 isolates inhibited the QS activity of *C. violaceum* CV026. The compounds in the extract might be damaging other components of the QS system, such as the stability and function of AHL, autoinducer synthase, and its regulators [26, 27].

Anti-QS agents in extracts of 1AC, 14PM, 16PM, 18PM, and CW17 isolates were likely proteins because higher absorbance value observed after proteinase-K and high-temperature treatment. The increasing absorbance value implied reduced anti-QS activity. The treatment might affect proteinaceous anti-QS compounds produced by actinobacteria, such as AHL-lactonase, acylase, decarboxylase, oxidoreductase, and deaminase [28], which the treatment can easily degrade. In the absence of anti-QS compounds, AHL will form protein-ligand complex, activating violacein production [29].

Extract from isolates 15PM, 20PM, CW01, and TB12 treated with proteinase-K showed higher anti-QS activity, indicating degradation of proteins that competitively bind to similar receptors or any proteins that might inhibit the anti-QS activity [30]. Additionally, anti-QS compounds might be a proenzyme or peptides hence the reaction with proteinase-K would positively affect the anti-QS activity. LaSarre reported actinobacteria produce proteinaceous compounds activated by serine proteases, enhancing anti-QS activity. [3, 8, 31].

Crude extracts had produced without fractionation and purification processes, potentially containing inhibitors or analogs that affect compound interactions. The interactions between undesirable compounds inevitably impact the effectiveness [32]. It might work antagonistically or synergistically, which could decrease or increase inhibition activity [31–33].

Biofilms consist of microorganisms and extracellular polymeric substances (EPSs). Disrupting the EPS layer might severely reduce biofilm formation. In *S. aureus*, the primary EPS components are polysaccharide intercellular adhesin, known as poly-N-acetyl-β-(1–6)-glucosamine, polysaccharide, proteins, and extracellular DNA (eDNA) [34]. *B. cereus* utilizes exopolysaccharides, proteins, and eDNA [35]. The EPSs of *E. faecalis* are predominantly eDNA and polysaccharides [36]. While *P. aeruginosa*’s EPS comprises eDNA, proteins, lipids, and polysaccharides, such as Psl, Pel, and alginate [37]. In *S.* Typhimurium, curli fimbriae and cellulose drive cell clustering and biofilm initiation [38]. In *V. cholerae* biofilms consist of vibrio-exopolysaccharides, proteins, and eDNA [39]. Based on previous researches [9, 40], Most of the isolates are belonging to the genera *Streptomyces* and *Arthrobacter*. *Streptomyces* produces ECM-degrading enzymes such as protease, amylase, nuclease, agarase, chitinase, and several organic acids such as docosanoic acid, tetracosanoic acid, arachidic acid, and erucic acid that are capable of inhibiting *P. aeruginosa* and *S. aureus* biofilms [41, 42]. *Arthrobacter* was reported to produce dextranase and xylanase, which may significantly eradicate the *P. aeruginosa*, methicillin-resistance *S. aureus*, and *E. coli* biofilms. Additionally, *Arthrobacter* produces glucuronide and cyclic-depsipeptides, such as arthroamide and turnagainolide, that could interfere the QS system [42, 43].

**Fig. 2:**
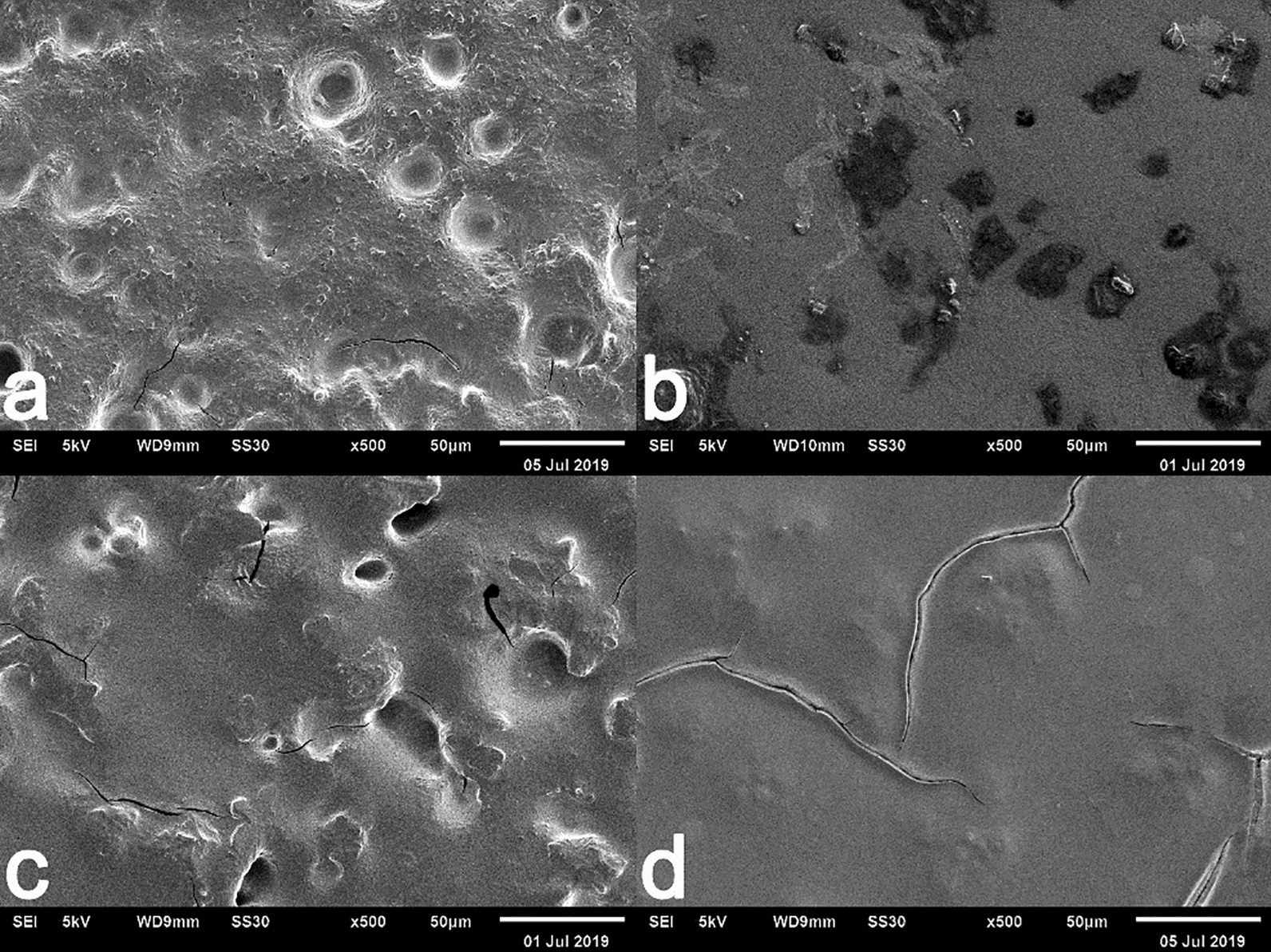
Biofilm of *B. cereus*. (**a**) without treatment (Control) (**b**) treated with crude extract of 20PM isolate. Biofilm of S. *Typhimurium*. (**c**) without treatment (Control) (**d**) treated with crude extract of 18PM isolate

Scanning electron microscopy showed the presence of *B. cereus* and *S.* Typhimurium biofilms. Antibiofilm activity can be observed due to the depletion and erosion across biofilm surfaces. Both extracts were competent to degrade the biofilm formation (Fig. 2) [44].

## Conclusions

In summary, this study showed the potential of actinobacterial extracts performing anti-QS activity against *C. violaceum* along with biofilm inhibition and elimination activities against biofilm-forming pathogens. They could be developed as a safer and more efficient disinfectants in several industries.

### Limitations

We did not determine the specific compound(s) responsible for antibiofilm and anti-QS activity and each isolate’s anti-QS mechanism. Furthermore, our characterization was limited to protein.

### Supplementary Information

**Additional file 1: Illustration S1.** Primary screening of anti-quorum sensing activity performed by 18PM Isolates against wild-type *C. violaceum *(ATCC 12472). **Illustration S2.** Ten actinobacteria isolates with positive anti-quorum sensing against wild-type *C. violaceum *(ATCC 12472). **Illustration S3.** Antimicrobial assay of actinobacterial crude extracts (50 μL, 10 mg/mL) against tested bacteria (a) *S. aureus *(b) *E. faecalis *(c) *B. cereus *(d) *V. cholerae *(e) *S. *Typhimurium (f) *P. aeruginosa *with K+: streptomycin (20 μL;10 mg/mL) as positive control and K-: DMSO (50 μL; 1%v/v) as negative control. **Illustration S4.** Secondary screening of actinobacterial crude extract of 18PM and 20PM (50 μL) (a) 5 mg/mL and (b) 10 mg/mL against wild-type *C. violaceum *(ATCC 12472) with K−: DMSO (50 μL; 1%v/v) as negative control.

## Data Availability

The datasets generated and/or analyzed during the current study are available from the corresponding author on reasonable request.

## Abbreviations

AHL: Acyl homoserine lactone
AMR: Antimicrobial resistance
ANOVA: Analysis of variance
BHIA: Brain heart infusion agar
BHIB: Brain heart infusion broth
ECM: Extracellular matrix
eDNA: Extracellular DNA
EPS: Extra polymeric substances
HHL: Hexanoyl-l-homoserine-lactone
LA: Luria agar
LB: Luria broth
QS: Quorum sensing
SEM: Scanning electron microscope
TSB: Tryptone soy broth
YMEA: Yeast malt extract agar
