# Supplementary material for: Control of pathogenic bacteria using marine actinobacterial extract with antiquorum sensing and antibiofilm activity
Source: BMC Res Notes. 2023 Nov 2;16:305. doi: 10.1186/s13104-023-06580-z (PMC10623884; doi:10.1186/s13104-023-06580-z)
Supplement: Supplementary file 1 — Additional file 1: Illustration S1. Primary screening of anti-quorum sensing activity performed by 18PM Isolates against wild-type C. violaceum (ATCC 12472). Illustration S2. Ten actinobacteria isolates with positive anti-quorum sensing against wild-type C. violaceum (ATCC 12472). Illustration S3. Antimicrobial assay of actinobacterial crude extracts (50 μL, 10 mg/mL) against tested bacteria (a) S. aureus (b) E. faecalis (c) B. cereus (d) V. cholerae (e) S. Typhimurium (f) P. aeruginosa with K+: streptomycin (20 μL;10 mg/mL) as positive control and K-: DMSO (50 μL; 1%v/v) as negative control. Illustration S4. Secondary screening of actinobacterial crude extract of 18PM and 20PM (50 μL) (a) 5 mg/mL and (b) 10 mg/mL against wild-type C. violaceum (ATCC 12472) with K−: DMSO (50 μL; 1%v/v) as negative control. [file 13104_2023_6580_MOESM1_ESM.pdf]

Additional file 1

Additional file 1 title: Supplementary illustrations

Additional file 1 descriptions: This file contains 3 figures and 1 table. All of them are referred on the main text of the manuscript as “Supplementary illustration S1, S2, S3, and S4”

Supplementary Illustration S1

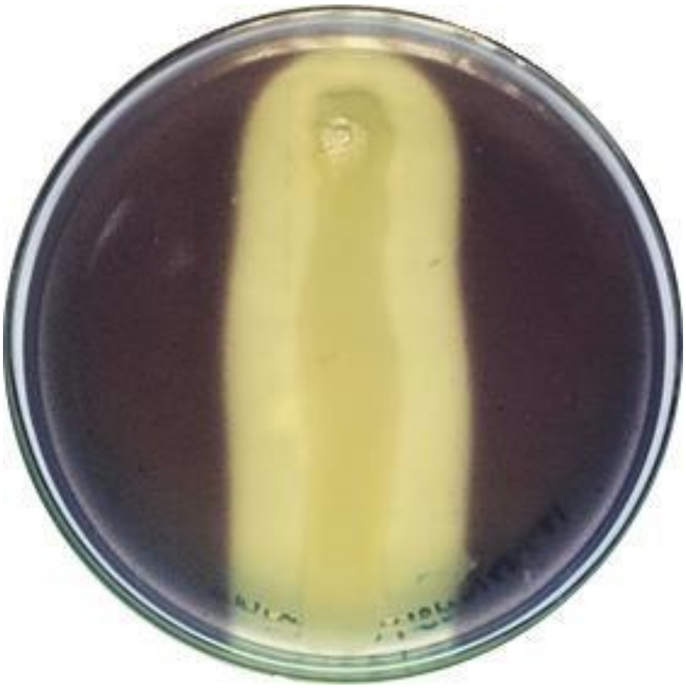

Additional file 1 Illustration S1 - Primary screening of anti-quorum sensing activity performed by 18PM Isolates against wild-type *C. violaceum* (ATCC 12472)

Supplementary Illustration 2

Additional file 1 Illustration S2 - Ten actinobacteria isolates with positive anti-quorum sensing against wild-type *C. violaceum* (ATCC 12472)

No	Isolate Code	Isolated From	Identified as
1	1AC	Pantai Ancol, North Jakarta, DKI Jakarta	-
2	11AC	Pantai Ancol, North Jakarta, DKI Jakarta	*Arthrobacter sp*
3	14PM	Pantai Mutiara, North Jakarta, DKI Jakarta	*Arthrobacter sp*
4	15PM	Pantai Mutiara, North Jakarta, DKI Jakarta	*Arthrobacter sp*
5	16PM	Pantai Mutiara, North Jakarta, DKI Jakarta	-
6	18PM	Pantai Mutiara, North Jakarta, DKI Jakarta	-
7	20PM	Pantai Mutiara, North Jakarta, DKI Jakarta	-
8	CW01	Cunca Wulang, West Manggarai, East Nusa Tenggara	*Arthrobacter sp*
9	CW17	Cunca Wulang, West Manggarai, East Nusa Tenggara	*Streptomyces sp.*
10	TB12	Telaga Biru, Tangerang, Banten	*Arthrobacter mysorens*

- Have not identified

Supplementary Illustration S3

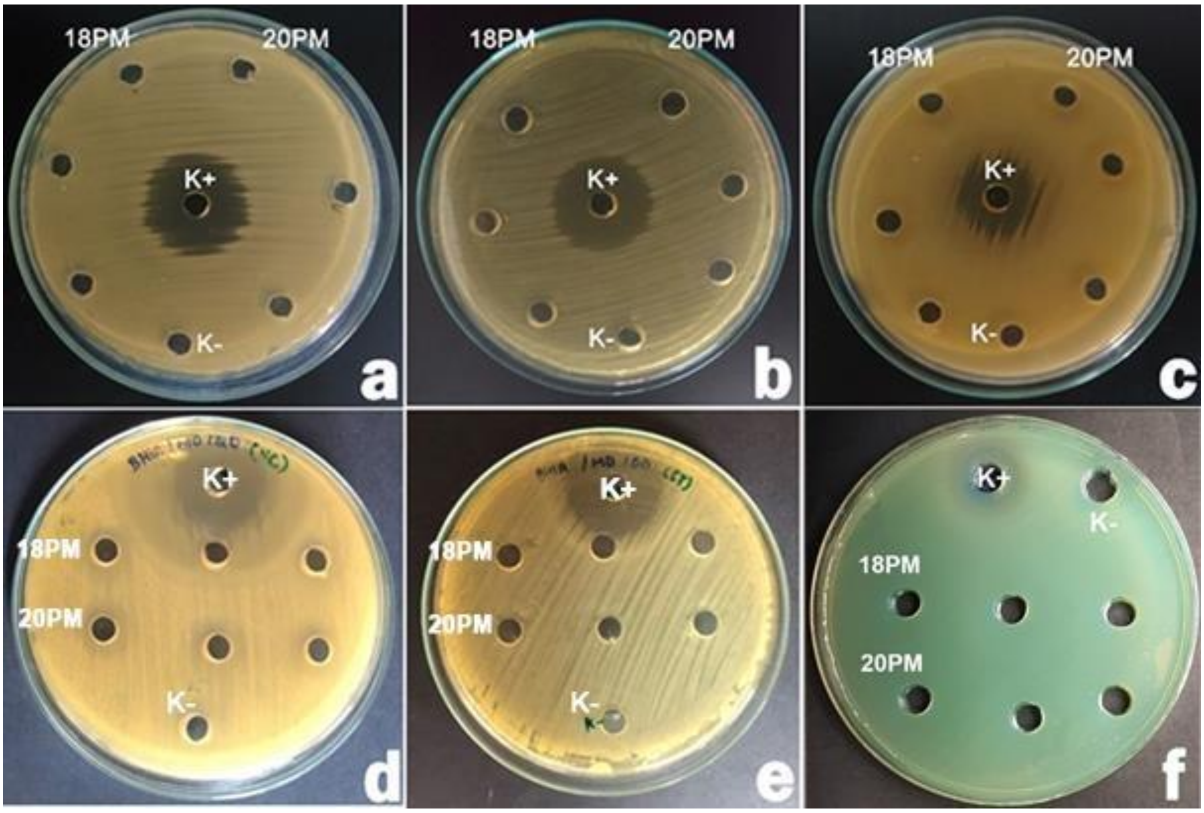

Additional file 1 Illustration S3 - Antimicrobial assay of actinobacterial crude extracts (50µL, 10 mg/mL) against tested bacteria (a) *S. aureus* (b) *E. faecalis* (c) *B. cereus* (d) *V. cholerae* (e) *S.* Typhimurium (f) *P. aeruginosa* with K+: streptomycin (20µL;10 mg/mL) as positive control and K-: DMSO (50µL; 1%v/v) as negative control.

Supplementary Illustration 4

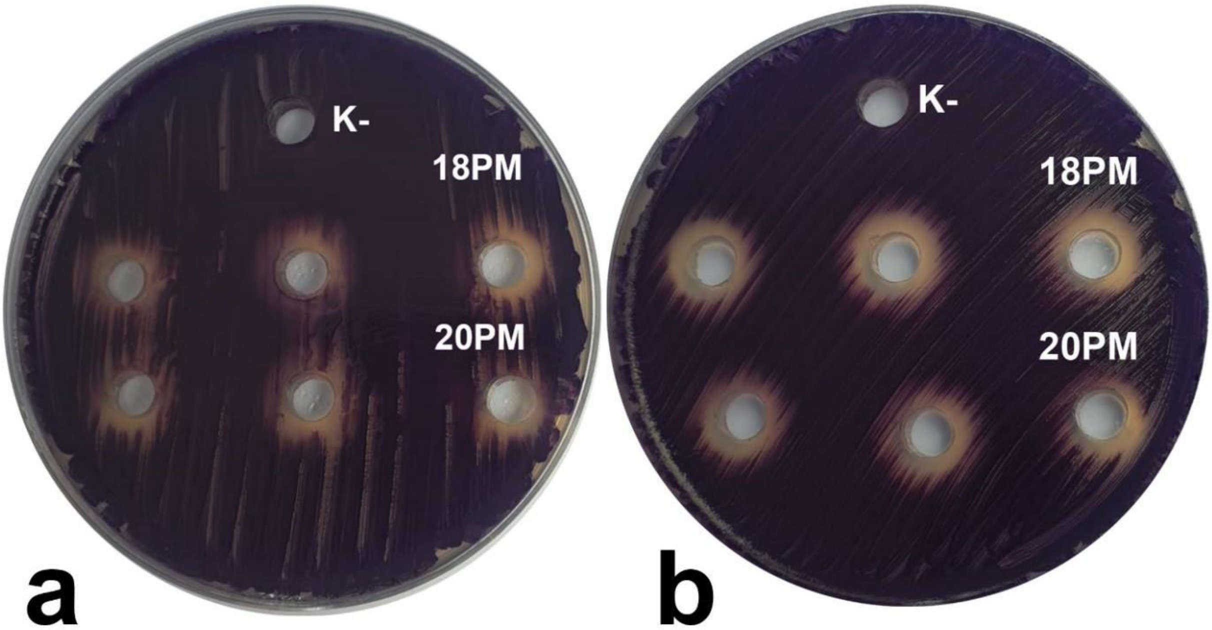

Additional file 1 Illustration S4 - Secondary screening of actinobacterial crude extract of 18PM and 20PM (50 µL) (a) 5mg/mL and (b) 10 mg/mL against wild-type *C. violaceum* (ATCC 12472) with K-: DMSO (50µL; 1%v/v) as negative control.
